# UCP2 and UCP3 variants and gene-environment interaction associated with prediabetes and T2DM in a rural population: a case control study in China

**DOI:** 10.1186/s12881-018-0554-4

**Published:** 2018-03-12

**Authors:** Meifang Su, Xiaoying Chen, Yue Chen, Congyun Wang, Songtao Li, Xuhua Ying, Tian Xiao, Na Wang, Qingwu Jiang, Chaowei Fu

**Affiliations:** 1 Yuhuan County Center for Disease Control and Prevention, Yuhuan, Zhejiang Province 317600 China; 2 School of Public Health, Fudan University, 138 Yi Xue Yuan Road, Xuhui District, Shanghai, 200032 China; 3 School of Epidemiology and Public Health, Faculty of Medicine, University of Ottawa, Ottawa, Ontario K1G 5Z3 Canada

**Keywords:** Type 2 diabetes, Prediabetes, Uncoupling protein gene, Gene polymorphism, Interaction, China

## Abstract

**Background:**

There are disparities for the association between uncoupling proteins (UCP) and type 2 diabetes (T2DM). The study was to examine the associations of genetic variants of UCP2 and UCP3 with prediabetes and T2DM in a rural Chinese population.

**Methods:**

A population-based case-control study of 397 adults with T2DM, 394 with prediabetes and 409 with normal glucose tolerance (NGT) was carried out in 2014 in a rural community in eastern China. Three groups were identified through a community survey and the prediabetes and NGT groups were frequently matched by age and gender with the T2DM group and they were not relatives of T2DM subjects. With r^2^ ≥ 0.8 and minor allele frequency (MAF) ≥0.05 for tag single nucleotide polymorphisms (SNPs) with potential function, three (rs660339, rs45560234 and rs643064) and six (rs7930460, rs15763, rs647126, rs1800849, rs3781907 and rs1685356) SNPs were selected respectively for UCP2 and UCP3 and genotyped in real time using the MassARRAY system (Sequenom; USA). The haplotypes, gene-environmental interaction and association between genetic variants of UCP2 and UCP3 and prediabetes or T2DM were explored.

**Results:**

There were no significant differences in age and sex among three study groups. After the adjustment for possible covariates, the A allele of rs1800849 in UCP3 was significantly associated with prediabetes (aOR_AA vs GG_ = 1.68, 95% CI: 1.02–2.78), and the association was also significant under the recessive model (aOR _AA vs GA + GG_ = 1.64, 95% CI: 1.02–2.66). Also, rs15763 was found to be marginally significantly associated with T2DM under dominant model (OR_GA + AA vs GG_ = 0.73, 95% CI: 0.52–1.03, *P* = 0.072). No haplotype was significantly associated with prediabetes or T2DM. Multiplicative interactions for rs660339-overweight on T2DM were observed. In addition, the AA genotype of rs660339 was associated with an increased risk of T2DM in overweight subjects (OR = 1.48, 95%CI: 0.87–2.52) but with a decreased risk in those with normal weight (OR = 0.54, 95%CI: 0.28–1.05).

**Conclusions:**

Rs1800849 in UCP3 was significantly associated with prediabetes. Overweight might modify the effects of rs660339 of UCP2 on T2DM.

**Electronic supplementary material:**

The online version of this article (10.1186/s12881-018-0554-4) contains supplementary material, which is available to authorized users.

## Background

Type 2 diabetes (T2DM) is characterized by chronic hyperglycemia caused by a combination of insulin resistance and inadequate compensatory insulin secretion. A recent study found that 11.6% of Chinese adults had diabetes and 50.1% had prediabetes, a precursor to T2DM [1]. T2DM is resulted from both genetic and environmental factors [2].

The uncoupling proteins (UCP) 2 and 3, members of the mitochondrial inner membrane carrier family [3], have been frequently studied for their roles in the development of T2DM. Although UCP2 and UCP3 share structural similarities, they have different tissue expressions in mammals [4]. UCP2 is distributed across a wide range of tissue and cell types, whereas UCP3 is mainly restricted to skeletal muscle and brown adipose tissue of humans [5–7]. UCP2 is an important regulator of mitochondrial oxidative stress damage induced by reactive oxygen species, and has been implicated with T2DM [8]. UCP3 is implicated in fatty acid (FA) metabolism, and patients with diabetes have low rather than high UCP3 protein content [9, 10]. But recent studies, including genome wide association studies (GWAS), have shown no evidence for any tagging-SNPs of UCP2-UCP3 tested that are associated with T2DM risk [11, 12], indicating disparities of association between UCP2 or UCP3 and T2DM. Although no associations were found between UCP2/UCP3 and diabetes in GWAS, some studies referred that peroxisome proliferator-activated receptor gamma (PPARG, related to diabetes confirmed in GWAS) interacted with Forkhead box protein O1 (FOXO1) to regulate the expression of UCP2 and rs659366 of UCP2 is unlikely to be identified through a GWAS strategy because it occurs predominantly in already diabetic subjects [12–14]. Little is known with regard to the association between UCP2/UCP3 polymorphisms and T2DM or prediabetes in Chinese population.

In recent years, some researchers have focused on the interaction between genetic and environmental factors (such as smoking, alcohol intake, physical activity level, general obesity, abdominal obesity, hypertension, dyslipidemia) for T2DM [15–17]. The interrelationship among adiposity, hypertension and UCP is likely complex. Several studies have found that UCP2 polymorphisms are associated with obesity [18–21] and hypertension [22]. Obesity and hypertension are associated with the development of insulin resistance, linked to the pathogenesis of T2DM [23, 24]. The exploration for the interactions between UCP polymorphisms and environmental factors can help for a better understanding of the association between UCP variants and T2DM.

In the current study, we used bioinformatics analyses to identify and select a set of variants in the UCP2/UCP3 region to determine the association between UCP2/UCP3 and prediabetes or T2DM in a Chinese population. We also examine the interactions of UCP2/UCP3 with adiposity and hypertension for prediabetes and T2DM.

## Methods

### Study design and subjects

The current study included 397 adults with T2DM, 394 with prediabetes and 409 with normal glucose tolerance (NGT), which was carried out in 2014 in a rural community in eastern China. The three groups of subjects were selected from participants of a cross-sectional study that was conducted during the period from June to December in 2012 in Yuhuan County, an island located in east China. That cross-sectional study was the baseline survey of the Yuhuan Rural Health Cohort Study, with a total 28,155 participants aged 20 years or older (born before 31 December, 1992), and collected information on demography, lifestyle (smoking, alcohol use, regular physical exercise) with a standard questionnaire and performed a free physical examination for those fasting for over 8 h. Venous blood of 5 ml were collected for fast plasma glucose (FPG) test, oral glucose tolerance test (OGTT) and blood lipids tests. Additional blood specimens were preserved under − 20°C. The Institutional Review Board of the Fudan University School of Public Health approved the study and all participants gave a written informed consent. The groups of newly diagnosed prediabetes and NGT subjects were frequently matched in age and sex with the T2DM group by excluding relatives. Subjects with T2DM were defined as those who had FPG ≥ 7.0 mmol/L or 2-h postload plasma glucose (2hPG) ≥ 11.1 mmol/L or having medical treatment history; subjects with prediabetes were defined as those who had 6.1 mmol/L ≤ FPG < 7.0 mmol/L or 7.8 mmol/L ≤ 2hPG < 11.1 mmol/L; NGT was defined as FPG < 6.1 mmol/L and 2hPG < 7.8 mmol/L [25]. Overweight was defined as body mass index (BMI) ≥ 25 kg/m^2^ [26].

### SNP selection and function prediction

A set of SNPs with potential function were selected using SNP Function Prediction of SNPinfo Web Server. According to HapMap dbSNP (CHB/JPT population) and NCBI dbSNP (Asian), a chromosomal from 3000 bp upstream to 3000 bp downstream of UCP2 and UCP3 was searched, and 12 SNPs (rs45560234, rs632862, rs659366, rs660339, rs15763, rs1800006, rs1800849, rs2075577, rs590336, rs647126, rs7926165, rs7930460) were obtained with potential function and minor allele frequency (MAF) > 0.05. Furtherly, linkage disequilibrium (LD) TAG SNP Selection was used to select haplotype-tagging SNPs (tag SNPs). With the same searched chromosomal range and NCBI dbSNP (Asian), r^2^ ≥ 0.8, MAF ≥ 0.05 for SNPs having potential function, rs660339 and rs1800849 as the mandatory entry site due to function prediction and previous studies [14–16], three SNPs of UCP2 (rs660339, rs45560234 and rs643064) and six SNPs of UCP3 (rs7930460, rs15763, rs647126, rs1800849, rs3781907 and rs1685356) were selected for this study (Additional file 1: Table S1).

### Genotyping

Genomic DNA used for polymerase chain reaction (PCR) amplification was extracted from peripheral blood using a DNA extraction kit (TIANGEN, China) according to the manufacturer’s instructions. Primers for amplification were designed with AssayDesigner 3.1 software. In the primer extension, ddH_2_O, 10X Buffer, 25 mM dNTP, 25 mM MgCl_2_, 0.5 μM Primer, 5 U/ul Hostar Taq and DNA template were used in the PCR reaction system. After purifying the products and transferring to SpectroCHIP, MALDI-TOF mass spectrometry was used for SNP genotyping. Thermocycling was carried out under the following conditions: Initial denaturation, 95 °C for 2 min followed by 45 cycles at 95 °C for 30 s (denaturation), 56 °C for 30 s (annealing), and 72 °C for 1 min (extension), with a final extension step at 72 °C for 5 min. The sequences of primers for amplification of the nine SNPs of UCP2–3 genes were presented in Additional file 2: Table S2. The MAFs of rs660339, rs45560234, rs643064, rs7930460, rs15763, rs647126, rs1800849, rs3781907 and rs1685356 in NGT group were 0.399 (A), 0.048 (A), 0.276 (T), 0.293 (G), 0.222 (A), 0.426 (G), 0.298 (A), 0.426 (G) and 0.392 (T), respectively.

### Statistical analysis

Data were described as mean ± standard deviation for continuous variables, and as frequencies (percentage) for non-continuous variables. Comparisons among the three groups were performed using one-way analyses of variance for continuous variables and χ^2^ test for categorical variables. Deviation from the Hardy-Weinberg equilibrium was assessed by χ^2^ test for the NGT group. Multinomial logistic regression analysis was performed to calculate crude odds ratios (ORs) and adjusted ORs (aORs) and their 95% confidential intervals (CIs). The genetic models tested in the study included additive, recessive and dominant models. Frequencies of haplotypes were calculated and compared among the three groups. Finally, we employed the general multifactor dimensionality reduction (GMDR) method [27] and logistic regression to estimate the interaction between gene and environmental risk factors.

Multinomial logistic regression, χ^2^ test and one-way analyses of variance were performed using SPSS version 19 software (Chicago, Illinois, USA). Haploview 4.2 and Hapstat3.0 were used to analyze linkage disequilibrium (LD) and haplotypes. GMDR software were used to estimate the interaction between gene and environmental factors. All tests were two-tailed, and it was considered statistically significant if a *p* value < 0.05 and marginally significant if a p value between 0.05 and 0.10. The Bonferroni for the number of SNPs was used to adjust the significance level of multiple comparisons when analyzing the association between genotypes and prediabetes or T2DM.

## Results

### Characteristics of the study population

The present study investigated 397 with newly diagnosed T2DM, 394 with newly diagnosed prediabetes and 409 who had NGT and three groups were matched by age and sex. Table 1 showed that those with T2DM or prediabetes were more likely to be hypertensive and overweight, and had lower income compared with those with NGT. No significant differences among three groups were found for age, sex, regular exercise, smoking and excessive drinking.

**Table 1:** General characteristics of subjects with normal glucose tolerance (NGT), prediabetes and type 2 diabetes (T2DM)

	NGT	Prediabetes	T2DM	χ^2^, *p* value
		Mean ± SD		
Age (years)	58.64 ± 11.62	58.73 ± 11.69	58.64 ± 11.82	0.008, 0.992^a^
		No. (%)		
Male	200 (48.90)	196 (49.75)	195 (49.12)	0.971, 0.615
Income (yuans)				6.025, 0.049
< 2000	203 (49.63)	179 (45.43)	215 (54.16)	
≥ 2000	206 (50.37)	215 (54.57)	182 (45.84)	
Regular exercise				2.223, 0.329
Yes	78 (19.07)	86 (21.83)	83 (20.91)	
No	331 (80.93)	308 (78.17)	314 (79.09)	
Smoking				1.946, 0.378
Yes	74 (18.09)	57 (14.47)	70 (17.63)	
No	335 (81.91)	337 (85.53)	327 (82.37)	
Excessive drinking				2.460, 0.292
Yes	36 (8.80)	46 (11.68)	47 (11.84)	
No	373 (91.20)	348 (88.32)	350 (88.16)	
Hypertension				20.666, < 0.001
Yes	124 (30.32)	148 (37.56)	182 (45.84)	
No	285 (69.68)	246 (62.44)	215 (54.16)	
BMI (kg/m^2^)				43.443, < 0.001
< 25	298 (72.86)	234 (59.39)	200 (50.38)	
≥ 25	111 (27.14)	160 (40.61)	197 (49.62)	

*NGT* normal glucose tolerance, *T2DM* type 2 diabetes, *BMI* body mass index, *SD* standard deviation

Bold denotes significant at *p*-value < 0.05

^a^One-way analyses of variance

### Associations of UCP2/UCP3 with prediabetes and T2DM

Table 2 and Additional file 3: Table S3 presented the distributions of three genotypes for each SNP and their associations with prediabetes or diabetes. All the genotype frequencies met the assumption of Hardy-Weinberg equilibrium for the NGT group (*p* > 0.05) except for rs15763. With respect to rs1800849 of UCP3, the genotype of AA (AA vs GG, *p* = 0.026; OR_AA vs GG_ = 1.75) was found to be a significant risk factor for prediabetes and after adjustment for age, sex, regular exercise, smoking, excessive drinking, hypertension and overweight, AA was still associated with an increased risk of prediabetes (aOR_AA vs GG_ = 1.68, 95% CI: 1.02–2.78); The recessive model also revealed a detrimental effect of AA for prediabetes (OR_AA vs GA + GG_ = 1.72, 95% CI: 1.07–2.76; aOR _AA vs GA + GG_ = 1.64, 95% CI: 1.02–2.66). In the recessive model, rs660339 (OR_AA vs GA + GG_ = 1.39, 95% CI: 0.96–2.00) and rs7930460 (OR_GG vs AG + AA_ = 1.52, 95%CI: 0.94–2.47) were marginally significantly associated with prediabetes, and A of rs660339 and G of rs7930460 tended to increase the risk of prediabetes. Additionally, rs15763 was found to be marginally significantly associated with T2DM in the dominant model (OR_GA + AA vs GG_ = 0.73, 95%CI: 0.52–1.03, *P* = 0.072) after adjusting for covariates, indicating that A could be a protective factor for T2DM (Table 2).

**Table 2:** Potential prediabetes or T2DM-associated UCP2 and UCP3 polymorphisms

Genotype	NGT	prediabetes	T2DM	p_HEW_	Prediabetes				T2DM			
					OR	p	aOR^a^	P^a^	OR	p	aOR^a^	p^a^
UCP2												
rs660339												
GG	142 (35.7)	136 (35.5)	132 (34.1)	0.751	1		1		1		1	
GA	194 (48.7)	169 (44.1)	191 (49.4)		0.91 (0.66,1.24)	0.553	0.90 (0.66,1.24)	0.534	1.06 (0.78,1.44)	0.716	1.05 (0.76,1.45)	0.763
AA	62 (15.6)	78 (20.4)	64 (16.5)		1.31 (0.87,1.98)	0.190	1.32 (0.87,2.00)	0.185	1.11 (0.73,1.69)	0.627	1.11 (0.72,1.71)	0.641
G	478 (60.1)	441 (57.6)	455 (58.8)									
Additive model (AA vs GA vs GG)					1.11 (0.91,1.35)	0.325	0.95 (0.63,1.43)	0.798	1.05 (0.86,1.29)	0.614	1.05 (0.86,1.29)	0.640
Recessive model (AA vs GA + GG)					1.39 (0.96,2.00)	**0.082**	1.40 (0.97,2.04)	**0.076**	1.07 (0.73,1.57)	0.714	1.08 (0.73,1.60)	0.710
Dominant model (GA + AA vs GG)					1.01 (0.75,1.35)	0.961	1.00 (0.75,1.35)	0.978	1.07 (0.80,1.44)	0.645	1.07 (0.79,1.44)	0.686
UCP3												
rs7930460												
AA	195 (49.1)	185 (48.1)	194 (49.4)	0.440	1		1		1		1	
AG	171 (43.1)	156 (40.5)	167 (42.5)		0.96 (0.72,1.29)	0.795	0.96 (0.71,1.29)	0.776	0.98 (0.73,1.31)	0.901	0.99 (0.73,1.34)	0.954
GG	31 (7.8)	44 (11.4)	32 (8.1)		1.50 (0.91,2.47)	0.116	1.43 (0.86,2.38)	0.173	1.04 (0.61,1.77)	0.892	1.02 (0.59,1.77)	0.939
A	561 (70.7)	526 (68.3)	555 (70.6)									
Additive model (GG vs AG vs AA)					1.12 (0.90,1.39)	0.315	1.10 (0.88,1.37)	0.403	1.00 (0.81,1.24)	0.985	1.00 (0.80,1.25)	0.990
Recessive model (GG vs AG + AA)					1.52 (0.94,2.47)	**0.087**	1.46 (0.89,2.38)	0.134	1.05 (0.63,1.75)	0.862	1.03 (0.60,1.75)	0.924
Dominant model (GG + AG vs AA)					1.04 (0.79,1.38)	0.765	1.03 (0.78,1.37)	0.839	0.99 (0.75,1.31)	0.945	1.00 (0.75,1.33)	0.974
rs15763												
GG	281 (72.8)	294 (77.8)	298 (78.0)	<0.001	1		1		1		1	
GA	39 (10.1)	32 (8.5)	29 (7.6)		0.78 (0.48,1.29)	0.336	0.783 (0.47,1.23)	0.342	0.70 (0.42,1.17)	0.170	0.67 (0.40,1.13)	0.136
AA	66 (17.1)	52 (13.8)	55 (14.4)		0.75 (0.51,1.12)	0.163	0.753 (0.50,1.13)	0.169	0.79 (0.53,1.16)	0.230	0.77 (0.51,1.15)	0.201
G	601 (77.8)	620 (82.0)	625 (81.8)									
Additive model (AA vs GA vs GG)					0.86 (0.71,1.04)	0.123	0.86 (0.71,1.05)	0.129	0.87 (0.72,1.05)	0.142	0.85 (0.70,1.04)	0.117
Recessive model (AA vs GA + GG)					0.77 (0.52,1.15)	0.202	0.77 (0.52,1.16)	0.210	0.82 (0.55,1.20)	0.305	0.80 (0.53,1.20)	0.279
Dominant model (GA + AA vs GG)					0.77 (0.55,1.06)	0.111	0.76 (0.55,1.07)	0.117	0.75 (0.54,1.05)	**0.094**	0.73 (0.52,1.03)	**0.072**
rs1800849												
GG	192 (48.2)	173 (44.7)	180 (45.7)	0.305	1		1		1		1	
GA	175 (44.0)	165 (42.6)	182 (46.2)		1.05 (0.78,1.41)	0.764	1.05 (0.78,1.41)	0.773	1.11 (0.83,1.48)	0.484	1.12 (0.83,1.52)	0.447
AA	31 (7.8)	49 (12.7)	32 (8.1)		1.75 (1.07,2.88)	**0.026**	1.68 (1.02,2.78)	**0.043**	1.10 (0.65,1.88)	0.724	1.06 (0.61,1.83)	0.842
G	559 (70.2)	511 (66.0)	542 (68.8)									
Additive model (AA vs GA vs GG)					1.22 (0.98,1.51)	**0.071**	1.20 (0.97,1.50)	0.098	1.07 (0.86,1.33)	0.527	1.07 (0.85,1.33)	0.581
Recessive model (AA vs GA + GG)					1.72 (1.07,2.76)	**0.025**	1.64 (1.02,2.66)	**0.043**	1.05 (0.63,1.75)	0.863	1.00 (0.59,1.70)	0.995
Dominant model (AA+GA vs GG)					1.15 (0.87,1.53)	0.320	1.14 (0.86,1.52)	0.361	1.11 (0.84,1.47)	0.471	1.11 (0.83,1.48)	0.473

*OR* odds ratio, *T2DM* type 2 diabetes, *UCP* uncoupling proteins

Bold denotes significant at *p*-value < 0.05 or marginal significant

^a^Adjusted for sex, age, regular exercise, income, excessive drinking, smoking, hypertension and overweight

### Linkage disequilibrium and haplotype analysis

There was a strong LD between rs643064 and rs660339 (r^2^ = 0.57) of UCP2 as well as between rs7930460 and rs647126 (r^2^ = 0.57), and between rs647126 and rs1685356 (r^2^ = 0.46–0.47). Combining the location information of SNPs, two haplotype blocks, that one included two SNPs (rs643064 and rs660339) of UCP2 and another included three SNPs (rs7930460, rs647126 and rs1685356) of UCP3, were detected. The frequencies of the haplotypes CG and CA formed by rs6413064-rs660339 of UCP2, AAT and AGC formed by rs7930460-rs647126-rs1685356 of UCP3 were lower, while the frequencies of TA formed by rs6413064-rs660339 of UCP2, GGC and AAC formed by rs7930460-rs647126-rs1685356 of UCP3 were higher in the prediabetes and T2DM groups compared to the NGT group. However, these haplotypes were not significantly associated with either prediabetes or T2DM in the regression analysis (Table 3).

**Table 3:** Associations of haplotypes with prediabetes and type 2 diabetes (T2DM)

Haplotype	Frequency (%)	OR	p	aOR^a^	p^a^
Prediabetes					
rs643064-rs660339					
CG	57.8	0.91 (0.74,1.12)	0.393	0.91 (0.74,1.12)	0.372
CA	12.0	0.95 (0.70,1.29)	0.736	0.98 (0.72,1.34)	0.907
TA	30.0	1.14 (0.91,1.43)	0.241	1.13 (0.90,1.42)	0.289
rs7930460-rs647126-rs1685356					
AAC	18.3	1.04 (0.80,1.35)	0.795	1.02 (0.78,1.33)	0.865
AAT	38.5	0.96 (0.78,1.18)	0.721	0.98 (0.79,1.21)	0.842
AGC	11.8	0.85 (0.62,1.15)	0.281	0.85 (0.63,1.16)	0.311
GGC	31.4	1.11 (0.89,1.38)	0.346	1.10 (0.88,1.37)	0.422
T2DM					
rs643064-rs660339					
CG	58.5	0.94 (0.77,1.15)	0.542	0.94 (0.76,1.16)	0.551
CA	11.5	0.90 (0.66,1.23)	0.514	0.90 (0.66,1.24)	0.533
TA	30.0	1.14 (0.92,1.43)	0.234	1.15 (0.91,1.44)	0.246
rs7930460-rs647126-rs1685356					
AAC	20.3	1.17 (0.91,1.51)	0.220	1.14 (0.87,1.48)	0.337
AAT	38.8	0.98 (0.80,1.20)	0.820	1.01 (0.81,1.24)	0.951
AGC	11.4	0.82 (0.60,1.11)	0.197	0.79 (0.58,1.09)	0.148
GGC	29.5	1.01 (0.81,1.26)	0.900	1.02 (0.81,1.28)	0.880

*OR* odds ratio, *T2DM* type 2 diabetes

^a^Adjusted for age, sex, regular exercise, income, excessive drinking, smoking, hypertension and overweight

### Gene-environmental interaction for prediabetes or T2DM

GMDR analysis was conducted to assess the interaction between the 9 SNPs and environment factors (income, regular exercise, smoking, excessive drinking, hypertension and overweight). Our GMDR analysis demonstrated that overweight was associated with prediabetes (testing accuracy = 56.1%, cross-validation consistency = 10/10, *P* = 0.011) and T2DM (testing accuracy = 60.9%, cross-validation consistency = 10/10, *P* = 0.001) in the one-factor model. There were potential interactions of overweight-rs1800849 on prediabetes (testing accuracy = 52.7%, cross-validation consistency = 10/10, P = 0.011) and of hypertension-overweight-rs660339 on T2DM (testing accuracy = 61.1%, cross-validation consistency = 10/10, *P* = 0.011) (Additional file 4: Table S4). We further tested the potential gene-environment interactions by logistic regression to validate the result of GMDR. Because in most cases recessive models showed a better fit for the association, we selected the recessive model in a further interaction analysis. We found that subjects with both overweight and AA of rs1800849 had an increased risk of prediabetes (OR = 3.92, 95% CI: 1.63–9.39), compared with an OR of 1.73 for those only with overweight and of 1.52 for those with only AA genotype, and the subjects with hypertension and overweight and AA of rs660339 also had an increased T2DM risk (OR = 7.78, 95% CI: 2.83–21.40) (Tables 4 and 5). Multiplicative interactions among rs660339, overweight and hypertension in association with T2DM were estimated in logistic regression analysis and only the interaction between rs660339 and overweight was significant (Table 5). Further stratified analysis revealed that the AA genotype of rs660339 was associated with an increased risk of T2DM for overweight subjects (OR = 1.48, 95% CI: 0.87–2.52) but with a decreased risk for those with normal weight (OR = 0.54, 95% CI: 0.28–1.05).

**Table 4:** Interaction analysis for rs1800849 and overweight on prediabetes

rs1800849	Overweight	NGT	Prediabetes	P	OR
GG/GA	No	265 (66.6)	203 (52.5)		1
GG/GA	Yes	102 (25.6)	135 (34.9)	**0.001**	1.73 (1.26,2.37)
AA	No	24 (6.0)	28 (7.2)	0.152	1.52 (0.86,2.71)
AA	Yes	7 (1.8)	21 (5.4)	**0.002**	3.92 (1.63,9.39)
Interaction				0.463	

*NGT* normal glucose tolerance, *OR* odds ratio

Bold denotes significant at *p*-value < 0.05

**Table 5:** Interaction analysis for rs660339 and hypertension and overweight on T2DM

rs660339	Overweight	Hypertension	NGT	T2DM	P	OR
GG/GA	No	No	181 (45.5)	93 (24.0)		1
GG/GA	No	Yes	59 (14.8)	77 (19.9)	**<0.001**	2.54 (1.67,3.87)
GG/GA	Yes	No	54 (13.6)	77 (19.9)	**<0.001**	2.78 (1.81,4.26)
GG/GA	Yes	Yes	42 (10.6)	76 (19.6)	**<0.001**	3.52 (2.24,5.54)
AA	No	No	33 (8.3)	16 (4.1)	0.861	0.94 (0.49,1.80)
AA	No	Yes	15 (3.8)	7 (1.8)	0.839	0.91 (0.36,2.31)
AA	Yes	No	9 (2.3)	21 (5.4)	**<0.001**	4.54 (2.00,10.31)
AA	Yes	Yes	5 (1.3)	20 (5.2)	**<0.001**	7.79 (2.83,21.40)
Interaction of rs660339-overweight					**0.021**	
Interaction of rs660339-hypertension					0.441	
Interaction of rs660339-hypertension-overweight					0.275	

*OR* odds ratio, *NGT* normal glucose tolerance, *T2DM* type 2 diabetes

Bold denotes significant at *p*-value < 0.05

## Discussion

In this study, three SNPs of UCP2 and six SNPs of UCP3 were genotyped and the associations between these SNPs and T2DM or prediabetes were evaluated in a rural Chinese population. The findings suggested a significant association between UCP3 rs1800849 and prediabetes in this population. No statistical significance was identified for the association between rs1800849 and T2DM. In previous studies, T allele was associated a lower incidence of T2DM in a French cohort [28], and rs1800849 was associated with elevated HDL-C levels and reduced BMI, independent of modifiable factors [29]. The discrepancies among studies may be due to ethnic differences in the genetic variation.

A number of previous studies have studied rs660339 and rs659366 of UCP2 associated with diabetes and the results are contradictory. G > A variation of rs660339 was found to be significantly associated with an increased risk of T2DM in Koreans [30], but with a decreased risk in Indians [31], while no significant association was found in Iranians and Europeans [32, 33]. de Souza BM et al. performed a comprehensive meta-analysis of 5 studies with a total of 2112 cases and 1841 controls, and demonstrated that rs660339 was significantly associated with an increased risk of T2DM only in Asian population [34]. No statistically significant association between rs660339 and prediabetes or T2DM was found in our study, which was consistent with results from another study of Chinese population [35].

Also, the interactions between hypertension, overweight and rs660339 and between overweight and rs1800849 were observed for T2DM and prediabetes, respectively. Logistic regression confirmed the multiplicative interaction of overweight-rs660339, and rs660339 might play a different role in populations with normal weight and overweight. UCP2 and UCP3 can play important roles in both fatty acid and blood sugar metabolism [8–10]. Our findings revealed that the AA genotype of rs660339 was a risk factor of T2DM for overweight subjects (OR = 1.48) but a protective factor for those with normal weight (OR = 0.54). Both UCP2 and overweight/obesity are associated with insulin resistance [21, 36, 37], a fundamental aspect of the etiology of T2DM. It is unknown however, why AA genotype of rs660339 influences T2DM differently in overweight and normal weight people. Previous studies demonstrated that TT of rs660339 increased the risk of overweight [20, 38], indicating that rs660339 could accelerate the development of prediabetes or T2DM through overweight. Another widely studied SNP of UCP2 was rs659366. Previous studies found that rs659366 was associated with decreased insulin sensitivity and obesity [21], and had a strong linkage disequilibrium with rs660339 with D’ = 0.946 and r^2^ = 0.657 [39], which were not observed in this study. Contradictory results had been reported concerning the association between overweight and rs1800849 [40–42].

To our knowledge, no studies have been published about the associations between rs647126 and rs1685356 and prediabetes or T2DM although GG of rs647126 and TT of rs1685356 were reported to be correlated with high BMI [43]. In previous studies of Fins and Caucasians [44, 45], rs15763 met the Hardy-Weinberg equilibrium, but not in the current population.

The current study was based on individuals with newly diagnosed T2DM and prediabetes. Potential gene-environmental interactions were evaluated using GMDR and confirmed by logistic regression. However, there were also some limitations. Firstly, the selection of SNPs was functional tagSNP strategy, and more studies were needed to confirm whether an association was due to SNPs themselves or the variants which were high LD with them. Secondly, genetic variants associated with prediabetes or T2DM were no longer significant after a Bonferroni correction (*P* < 0.05/9). Although Bonferroni correction was too strict generally, a larger size sample was needed to confirm the reliability of the associations. Additionally, covariates including lifestyle factors, age and sex had been adjusted but known genetic variants such as TCF7L2 and KCNJ11 had not been tested and controlled in this study.

## Conclusion

In conclusion, rs1800849 in UCP3 were significantly associated with prediabetes in a rural Chinese population. Overweight modified the effect of rs660339 of UCP2 on T2DM. These findings suggested that rs1800849 in UCP3 and rs660339 in UCP2 might play an important role in the incidence and development of T2DM.

## Additional files

Additional file 1: **Table S1**. Main information of tSNPs and their function prediction. (DOCX 17 kb)

Additional file 2: **Table S2**. Amplification and extension primers sequences of the nine loci in UCP2–3 genes. (DOCX 16 kb)

Additional file 3: **Table S3**. Associations between UCP2, UCP3 and prediabetes or T2DM. (DOCX 31 kb)

Additional file 4: **Table S4**. Best gene-environment interaction models identified by GMDR. (DOCX 16 kb)

## Abbreviations

2hPG: 2-h postload plasma glucose
BMI: Body mass index
FPG: Fast plasma glucose
GMDR: General multifactor dimensionality reduction
GWAS: Genome wide association study
LD: Linkage disequilibrium
NGT: Normal glucose tolerance
OGTT: Oral glucose tolerance test
PCR: Polymerase chain reaction
SNPs: Single nucleotide polymorphisms
T2DM: Type 2 diabetes mellitus
UCP: Uncoupling protein

## References

[1] Xu Y, Wang L, He J, Bi Y, Li M, Wang T (2013). Prevalence and control of diabetes in Chinese adults. JAMA.

[2] Samsom M, Trivedi T, Orekoya O, Vyas S (2016). Understanding the importance of gene and environment in the etiology and prevention of type 2 diabetes mellitus in high-risk populations. Oral Health Case Rep.

[3] Diehl AM, Hoek JB (1999). Mitochondrial uncoupling: role of uncoupling protein anion carriers and relationship to thermogenesis and weight control “the benefits of losing control”. J Bioenerg Biomembr.

[4] Erlanson-Albertsson C (2002). Uncoupling proteins--a new family of proteins with unknown function. Nutr Neurosci.

[5] Souza BM, Assmann TS, Kliemann LM, Gross JL, Canani LH, Crispim D (2011). The role of uncoupling protein 2 (UCP2) on the development of type 2 diabetes mellitus and its chronic complications. Arq Bras Endocrinol Metabol.

[6] Dalgaard LT, Pedersen O (2001). Uncoupling proteins: functional characteristics and role in the pathogenesis of obesity and type II diabetes. Diabetologia.

[7] Boss O, Samec S, Paoloni-Giacobino A, Rossier C, Dulloo A, Seydoux J (1997). Uncoupling protein-3: a new member of the mitochondrial carrier family with tissue-specific expression. FEBS Lett.

[8] Casteilla L, Rigoulet M, Pénicaud L (2001). Mitochondrial ROS metabolism: modulation by uncoupling proteins. IUBMB Life.

[9] Schrauwen P, Hoeks J, Hesselink MK (2006). Putative function and physiological relevance of the mitochondrial uncoupling protein-3: involvement in fatty acid metabolism?. Prog Lipid Res.

[10] Schrauwen P, Mensink M, Schaart G, Moonen-Kornips E, Sels JP, Blaak EE (2006). Reduced skeletal muscle uncoupling protein-3 content in prediabetic subjects and type 2 diabetic patients: restoration by rosiglitazone treatment. J Clin Endocrinol Metab.

[11] Zee RY, Ridker PM, Chasman DI (2011). Mitochondrial uncoupling protein gene cluster variation (UCP2-UCP3) and the risk of incident type 2 diabetes mellitus: the women’s genome health study. Atherosclerosis.

[12] GWAS catalog. http://www.ebi.ac.uk/gwas/. Accessed 2 Mar 2018.

[13] Dalgaard LT (2011). Genetic variance in uncoupling protein 2 in relation to obesity, type 2 diabetes, and related metabolic traits: focus on the functional −866G>a promoter variant (rs659366). J Obes.

[14] Wu C, Gong Y, Yuan J, Gong H, Zou Y, Ge J (2012). Identification of shared genetic susceptibility locus for coronary artery disease, type 2 diabetes and obesity: a meta-analysis of genome-wide studies. Cardiovasc Diabetol.

[15] Zhang L, Wang J, Zhang M, Wang G, Shen Y, Wu D (2017). Association of type 2 diabetes mellitus with the interaction between low-density lipoprotein receptor-related protein 5 (LRP5) polymorphisms and overweight and obesity in rural Chinese adults. J Diabetes.

[16] Perry JR, Voight BF, Yengo L, Amin N, Dupuis J, Ganser M (2012). Stratifying type 2 diabetes cases by BMI identifies genetic risk variants in LAMA1 and enrichment for risk variants in lean compared to obese cases. PLoS Genet.

[17] Liu L, Chen L, Li Z, Li L, Wang M, Qu J (2015). Association of genetic variants in TOMM7 gene and gene environment interaction with type 2 diabetes in Chinese Dong population. Zhong Nan Da Xue Xue Bao Yi Xue Ban.

[18] Gul A, Ateş Ö, Özer S, Kasap T, Ensari E, Demir O (2017). Role of the polymorphisms of uncoupling protein genes in childhood obesity and their association with obesity-related disturbances. Genet Test Mol Biomarkers.

[19] Brondani LA, de Souza BM, Assmann TS, Bouças AP, Bauer AC, Canani LH (2014). Association of the UCP polymorphisms with susceptibility to obesity: case-control study and meta-analysis. Mol Biol Rep.

[20] Zhang M, Wang M, Zhao ZT (2014). Uncoupling protein 2 gene polymorphisms in association with overweight and obesity susceptibility: a meta-analysis. Meta Gene.

[21] Andersen G, Dalgaard LT, Justesen JM, Anthonsen S, Nielsen T, Thørner LW (2013). The frequent UCP2-866G>A polymorphism protects against insulin resistance and is associated with obesity: a study of obesity and related metabolic traits among 17 636 Danes. Int J Obes.

[22] Ji Q, Ikegami H, Fujisawa T, Kawabata Y, Ono M, Nishino M (2004). A common polymorphism of uncoupling protein 2 gene is associated with hypertension. J Hypertens.

[23] Kahn BB, Flier JS (2000). Obesity and insulin resistance. J Clin Invest.

[24] Reaven GM (1995). Pathophysiology of insulin resistance in human disease. Physiol Rev.

[25] Alberti KG, Zimmet PZ (1998). Definition, diagnosis and classification of diabetes mellitus and its complications. Part 1: diagnosis and classification of diabetes mellitus. Provisional report of a WHO consultation. Diabet Med.

[26] American Medical Association (1998). Executive summary of the clinical guidelines on the identification, evaluation, and treatment of overweight and obesity in adults. Arch Intern Med..

[27] Xu HM, Xu LF, Hou TT, Luo LF, Chen GB, Sun XW (2016). GMDR: versatile software for detecting gene-gene and gene-environment interactions underlying complex traits. Curr Genomics.

[28] Meirhaeghe A, Amouyel P, Helbecque N, Cottel D, Otabe S, Froguel P (2000). An uncoupling protein 3 gene polymorphism associated with a lower risk of developing type II diabetes and with atherogenic lipid profile in a French cohort. Diabetologia.

[29] Hamada T, Kotani K, Fujiwara S, Sano Y, Domichi M, Tsuzaki K (2008). The common-55 C/T polymorphism in the promoter region of the uncoupling protein 3 gene reduces prevalence of obesity and elevates serum high-density lipoprotein cholesterol levels in the general Japanese population. Metabolism.

[30] Lee HJ, Ryu HJ, Shin HD, Park BL, Kim JY, Cho YM (2008). Associations between polymorphisms in the mitochondrial uncoupling proteins (UCPs) with T2DM. Clin Chim Acta.

[31] Vimaleswaran KS, Radha V, Ghosh S, Majumder PP, Sathyanarayana Rao MR, Mohan V (2011). Uncoupling protein 2 and 3 gene polymorphisms and their association with type 2 diabetes in Asian Indians. Diabetes Technol Ther.

[32] Heidari J, Akrami SM, Heshmat R, Amiri P, Fakhrzadeh H, Pajouhi M (2010). Association study of the-866G/A UCP2 gene promoter polymorphism with type 2 diabetes and obesity in a Tehran population: a case control study. Arch Iran Med.

[33] Bielinski SJ, Pankow JS, Boerwinkle E, Bray MS, Kao WH, Folsom AR (2008). Lack of association between uncoupling protein-2 Ala55Val polymorphism and incident diabetes in the atherosclerosis risk in communities study. Acta Diabetol.

[34] de Souza BM, Brondani LA, Boucas AP, Sortica DA, Kramer CK, Canani LH (2013). Associations between UCP1-3826A/G, UCP2-866G/A, Ala55Val and Ins/Del, and UCP3-55C/T polymorphisms and susceptibility to type 2 diabetes mellitus: case-control study and meta-analysis. PLoS One.

[35] Shen Y, Wen Z, Wang N, Zheng Z, Liu K, Xia X (2014). Investigation of variants in UCP2 in Chinese type 2 diabetes and diabetic retinopathy. PLoS One.

[36] Chan CB, De Leo D, Joseph JW, McQuaid TS, Ha XF, Xu F (2001). Increased uncoupling protein-2 levels in beta-cells are associated with impaired glucose-stimulated insulin secretion mechanism of action. Diabetes.

[37] Kim JH, Li L, Yun JH, Choi BC, Baek KH (2011). Association study between the -866G/A polymorphism in the promoter of uncoupling protein-2 gene and polycystic ovary syndrome. Mol Med Rep.

[38] Martinez-Hervas S, Mansego ML, de Marco G, Martinez F, Alonso MP, Morcillo S (2012). Polymorphisms of the UCP2 gene are associated with body fat distribution and risk of abdominal obesity in Spanish population. Eur J Clin Investig.

[39] Oktavianthi S, Trimarsanto H, Febinia CA, Suastika K, Saraswati MR, Dwipayana P (2012). Uncoupling protein 2 gene polymorphisms are associated with obesity. Cardiovasc Diabetol.

[40] de Luis DA, Aller R, Izaola O, Romero E (2016). Effect of -55CT polymorphism of UCP3 on insulin resistance and cardiovascular risk factors after a high protein/low carbohydrate versus a standard hypocaloric diet. Ann Nutr Metab.

[41] Xu YP, Liang L, Wang CL, Fu JF, Liu PN, Lv LQ (2013). Association between UCP3 gene polymorphisms and nonalcoholic fatty liver disease in Chinese children. World J Gastroenterol.

[42] Zou Z, Mao L, Shi Y, Chen J, Wang L, Cai W (2017). Association between peroxisome proliferator-activated receptor, UCP3 and lipoprotein lipase gene polymorphisms and obesity in Chinese adolescents. Obes Res Clin Pract.

[43] van Abeelen AF, de Krom M, Hendriks J, Grobbee DE, Adan RA, van der Schouw YT (2008). Variations in the uncoupling protein-3 gene are associated with specific obesity phenotypes. Eur J Endocrinol.

[44] Salopuro T, Pulkkinen L, Lindström J, Kolehmainen M, Tolppanen AM, Eriksson JG (2009). Variation in the UCP2 and UCP3 genes associates with abdominal obesity and serum lipids: the finnish diabetes prevention study. BMC Med Genet.

[45] Acosta A, Camilleri M, Shin A, Vazquez-Roque MI, Iturrino J, Lanza IR (2015). Association of UCP-3 rs1626521 with obesity and stomach functions in humans. Obesity (Silver Spring).

