# Supplementary material for: UCP2 and UCP3 variants and gene-environment interaction associated with prediabetes and T2DM in a rural population: a case control study in China
Source: BMC Med Genet. 2018 Mar 12;19:43. doi: 10.1186/s12881-018-0554-4 (PMC5848510; doi:10.1186/s12881-018-0554-4)
Supplement: Supplementary file 1 — Table S1. Main information of tSNPs and their function prediction. (DOCX 17 kb) [file 12881_2018_554_MOESM1_ESM.docx]

| **Table S1** Main information of tSNPs and their function prediction | | | | | | | | | | | | |
| --- | --- | --- | --- | --- | --- | --- | --- | --- | --- | --- | --- | --- |
| Gene | SNP | Position | Function | Allele | TFBS | Splicing(ESE or ESS) | miRNA(miRanda) | miRNA(Sanger) | nsSNP | Polyphen | RegPotential | Conservation |
| UCP2 | rs643064 | 11:73973670 | downstream variant | T/C | -- | -- | -- | -- | -- | -- | 0.051356 | 0 |
| UCP2 | rs660339 | 11:73978059 | Missense | G/A | -- | Y | -- | -- | Y | benign | 0.420954 | 0 |
| UCP2 | rs45560234 | 11:73982793 | utr variant 5 prime | G/A | Y | -- | -- | -- | -- | -- | NA | NA |
| UCP3 | rs7930460 | 11:73999841 | downstream variant 500B | A/G | Y | -- | -- | -- | -- | -- | NA | 0 |
| UCP3 | rs15763 | 11:74000432 | utr variant 3 prime | G/A | Y | -- | Y | -- | -- | -- | 0.048432 | 0 |
| UCP3 | rs647126 | 11:74000975 | utr variant 3 prime | A/G | Y | -- | Y | Y | -- | -- | 0.232779 | 0 |
| UCP3 | rs1685356 | 11:74001814 | intron variant | T/C | -- | -- | -- | -- | -- | -- | 0.11093 | 0 |
| UCP3 | rs3781907 | 11:74005424 | intron variant | G/A | -- | -- | -- | -- | -- | -- | 0 | 0.011 |
| UCP3 | rs1800849 | 11:74009120 | utr variant 5 prime | G/A | Y | -- | -- | -- | -- | -- | 0.154132 | 0.037 |
