# Supplementary material for: UCP2 and UCP3 variants and gene-environment interaction associated with prediabetes and T2DM in a rural population: a case control study in China
Source: BMC Med Genet. 2018 Mar 12;19:43. doi: 10.1186/s12881-018-0554-4 (PMC5848510; doi:10.1186/s12881-018-0554-4)
Supplement: Supplementary file 2 — Table S2. Amplification and extension primers sequences of the nine loci in UCP2–3 genes. (DOCX 16 kb) [file 12881_2018_554_MOESM2_ESM.docx]

| **Table S2** Amplification and extension primers sequences of the nine loci in UCP2-3 genes | | | |
| --- | --- | --- | --- |
| SNPs | Forward Primer | Reverse Primer | Extension Primer |
| rs643064 | ACGTTGGATGAAGAACAGGGACAGAGAAGC | ACGTTGGATGTGCCCCAGCTAGGCATCCAA | CAAGGCCCTGGCTTCTTGT |
| rs660339 | ACGTTGGATGTGGTCAGAATGGTGCCCATC | ACGTTGGATGGATCCAAGGAGAAAGTCAGG | CGCGGTACTGGGCGCTGG |
| rs45560234 | ACGTTGGATGCCTTGGGATTGACTGTCCAC | ACGTTGGATGTGCGTGCGGCTGTGTCTGT | GACTGTCCACGCTCGCCT |
| rs7930460 | ACGTTGGATGCACACCACATGTCTGCTTTC | ACGTTGGATGAACAATCGCTGCTTCACAGG | ctccTTCCAAGGAGTTTTAGGTCAG |
| rs15763 | ACGTTGGATGATGACGTGAAGCCACAGGAG | ACGTTGGATGAAGCCCAACAATCCTTTGAG | gaaCCACCCCCCCCACCACGT |
| rs647126 | ACGTTGGATGATGCATGTGTGATGGTGCAG | ACGTTGGATGACAATGGAGTGGGAGATCAG | cGCTCTGTGTTGCTGGGTGGCG |
| rs1685356 | ACGTTGGATGTTGAGCTCTTAACCTCCCTG | ACGTTGGATGGCAGCGAATTCTCACAGTTC | caaGCCTCAGTTTGTGAAAAG |
| rs3781907 | ACGTTGGATGTTAGGTGTCACCTTCTCTGC | ACGTTGGATGAAGATGCAGCTACACCACAG | CCTCTGGGGCAACCCCTG |
| rs1800849 | ACGTTGGATGAGGCTGTCAACCAACTTCTC | ACGTTGGATGTGTTGTCTCTGCTGCTTCTG | ccccTAAGGTTTCAGGTCAGCCT |
