# Supplementary material for: UCP2 and UCP3 variants and gene-environment interaction associated with prediabetes and T2DM in a rural population: a case control study in China
Source: BMC Med Genet. 2018 Mar 12;19:43. doi: 10.1186/s12881-018-0554-4 (PMC5848510; doi:10.1186/s12881-018-0554-4)
Supplement: Supplementary file 3 — Table S3. Associations between UCP2, UCP3 and prediabetes or T2DM. (DOCX 31 kb) [file 12881_2018_554_MOESM3_ESM.docx]

| **Table S3** Associations between UCP2, UCP3 and prediabetes or T2DM | | | | | | | | | | | | | |
| --- | --- | --- | --- | --- | --- | --- | --- | --- | --- | --- | --- | --- | --- |
| Genotype | NGT | prediabetes | T2DM | P_HEW_ | Prediabetes | | | |  | T2DM | | |  |
|  |  |  |  |  | OR | P | aOR* | P* |  | OR | P | aOR* | P* |
| UCP2 |  |  |  |  |  |  |  |  |  |  |  |  |  |
| rs643064 |  |  |  |  |  |  |  |  |  |  |  |  |  |
| CC | 200(51.8) | 188(50.1) | 184(48.5) | 0.543 | 1 |  | 1 |  |  | 1 |  | 1 |  |
| CT | 159(41.2) | 152(40.5) | 161(42.5) |  | 1.017(0.755,1.371) | 0.912 | 1.020(0.754,1.379) | 0.898 |  | 1.101(0.818,1.481) | 0.527 | 1.117(0.822,1.517) | 0.479 |
| TT | 27(7.0) | 35(9.3) | 34(9.0) |  | 1.379(0.804,2.367) | 0.243 | 1.322(0.763,2.291) | 0.319 |  | 1.369(0.795,2.357) | 0.258 | 1.350(0.769,2.371) | 0.296 |
| C | 559(72.4) | 528(70.4) | 529(69.8) |  |  |  |  |  |  |  |  |  |  |
| Additive model  (TT vs CT vs CC) |  |  |  |  | 1.104(0.883,1.379) | 0.385 | 1.092(0.870,1.369) | 0.448 |  | 1.137(0.911,1.419) | 0.258 | 1.139(0.905,1.434) | 0.266 |
| Recessive model  (TT vs CT+CC) |  |  |  |  | 1.369(0.811,2.310) | 0.240 | 1.311(0.769,2.232) | 0.319 |  | 1.310(0.774,2.218) | 0.314 | 1.284(0.744,2.214) | 0.370 |
| Dominant model  (CT+TT vs CC) |  |  |  |  | 1.070(0.805,1.421) | 0.643 | 1.064(0.798,1.419) | 0.674 |  | 1.140(0.858,1.513) | 0.367 | 1.150(0.858,1.542) | 0.348 |
| rs45560234 |  |  |  |  |  |  |  |  |  |  |  |  |  |
| GG | 349(90.6) | 331(88.0) | 348(89.9) | 0.901 | 1 |  | 1 |  |  | 1 |  | 1 |  |
| GA | 35(9.1) | 44(11.7) | 39(10.1) |  | 1.326(0.830,2.118) | 0.239 | 1.396(0.868,2.244) | 0.169 |  | 1.117(0.692,1.806) | 0.650 | 1.159(0.707,1.900) | 0.558 |
| AA | 1(0.3) | 1(0.3) | 0(0.0) |  | 1.054(0.066,16.926) | 0.970 | 1.058(0.055,20.382) | 0.970 |  | - | - | - | - |
| G | 733(95.2) | 706(93.9) | 735(95.0) |  |  |  |  |  |  |  |  |  |  |
| Additive model  (AA vs GA vs GG) |  |  |  |  | 1.298(0.828,2.037) | 0.256 | 1.363(0.863,2.155) | 0.184 |  | 1.052(0.660,1.677) | 0.830 | 1.082(0.670,1.748) | 0.748 |
| Recessive model  (AA vs GA+GG) |  |  |  |  | 1.024(0.064,16.431) | 0.987 | 1.016(0.053,19.540) | 0.992 |  | - | - | - | - |
| Dominant model  (AA+GA vs GG) |  |  |  |  | 1.318(0.829,2.095) | 0.243 | 1.386(0.867,2.218) | 0.173 |  | 1.086(0.674,1.750) | 0.733 | 1.123(0.687,1.834) | 0.644 |
| UCP3 |  |  |  |  |  |  |  |  |  |  |  |  |  |
| rs647126 |  |  |  |  |  |  |  |  |  |  |  |  |  |
| AA | 127(32.4) | 134(34.7) | 137(34.9) | 0.658 | 1 |  | 1 |  |  | 1 |  | 1 |  |
| AG | 196(50.0) | 169(43.8) | 189(48.2) |  | 0.817(0.594,1.123) | 0.214 | 0.816(0.592,1.127) | 0.217 |  | 0.894(0.653,1.223) | 0.483 | 0.912(0.660,1.261) | 0.578 |
| GG | 69(17.6) | 83(21.5) | 66(16.8) |  | 1.140(0.763,1.703) | 0.522 | 1.117(0.743,1.679) | 0.595 |  | 0.887(0.586,1.343) | 0.570 | 0.862(0.562,1.324) | 0.498 |
| A | 450(57.4) | 437(56.6) | 463(59.1) |  |  |  |  |  |  |  |  |  |  |
| Additive model  (GG vs AG vs AA) |  |  |  |  | 1.032(0.847,1.258) | 0.756 | 1.022(0.836,1.250) | 0.831 |  | 0.936(0.768,1.141) | 0.512 | 0.928(0.756,1.138) | 0.472 |
| Recessive model  (GG vs AG+AA) |  |  |  |  | 1.282(0.898,1.830) | 0.171 | 1.257(0.876,1.805) | 0.215 |  | 0.948(0.654,1.373) | 0.777 | 0.911(0.621,1.336) | 0.632 |
| Dominant model  (GG+AG vs AA) |  |  |  |  | 0.901(0.669,1.214) | 0.494 | 0.895(0.662,1.210) | 0.471 |  | 0.892(0.663,1.200) | 0.450 | 0.898(0.661,1.219) | 0.491 |
| rs1685356 |  |  |  |  |  |  |  |  |  |  |  |  |  |
| CC | 142(35.8) | 146(38.0) | 140(35.8) | 0.301 | 1 |  | 1 |  |  | 1 |  | 1 |  |
| CT | 199(50.1) | 179(46.6) | 196(50.1) |  | 0.875(0.644,1.189) | 0.393 | 0.874(0.640,1.193) | 0.397 |  | 0.999(0.736,1.356) | 0.995 | 0.996(0.726,1.365) | 0.979 |
| TT | 56(14.1) | 59(15.4) | 55(14.1) |  | 1.025(0.665,1.579) | 0.912 | 1.060(0.683,1.644) | 0.796 |  | 0.996(0.642,1.546) | 0.986 | 1.072(0.681,1.687) | 0.765 |
| C | 483(60.8) | 471(61.3) | 476(60.9) |  |  |  |  |  |  |  |  |  |  |
| Additive model  (TT vs CT vs CC) |  |  |  |  | 0.979(0.796,1.203) | 0.838 | 0.991(0.804,1.222) | 0.934 |  | 0.998(0.813,1.226) | 0.987 | 1.026(0.830,1.269) | 0.813 |
| Recessive model  (TT vs CT+CC) |  |  |  |  | 1.105(0.744,1.642) | 0.620 | 1.144(0.765,1.710) | 0.512 |  | 0.997(0.667,1.489) | 0.987 | 1.074(0.710,1.626) | 0.735 |
| Dominant model  (TT+CT vs CC) |  |  |  |  | 0.908(0.679,1.214) | 0.514 | 0.914(0.680,1.228) | 0.551 |  | 0.998(0.746,1.336) | 0.991 | 1.012(0.749,1.368) | 0.936 |
| rs3781907 |  |  |  |  |  |  |  |  |  |  |  |  |  |
| AA | 124(31.2) | 124(32.5) | 135(35.0) | 0.141 | 1 |  | 1 |  |  | 1 |  | 1 |  |
| AG | 209(52.5) | 184(48.2) | 178(46.1) |  | 0.880(0.640,1.210) | 0.433 | 0.860(0.622,1.188) | 0.359 |  | 0.782(0.571,1.072) | 0.127 | 0.764(0.551,1.060) | 0.107 |
| GG | 65(16.3) | 74(19.4) | 73(18.9) |  | 1.138(0.751,1.726) | 0.541 | 1.134(0.744,1.729) | 0.560 |  | 1.032(0.682,1.560) | 0.883 | 1.070(0.698,1.640) | 0.757 |
| A | 457(57.4) | 432(56.5) | 448(58.0) |  |  |  |  |  |  |  |  |  |  |
| Additive model  (GG vs AG vs AA) |  |  |  |  | 1.036(0.848,1.266) | 0.729 | 1.031(0.841,1.264) | 0.766 |  | 0.975(0.798,1.191) | 0.804 | 0.987(0.802,1.215) | 0.904 |
| Recessive model  (GG vs AG+AA) |  |  |  |  | 1.231(0.852,1.777) | 0.268 | 1.244(0.857,1.805) | 0.251 |  | 1.195(0.827,1.727) | 0.343 | 1.257(0.860,1.837) | 0.238 |
| Dominant model  (GG+AG vs AA) |  |  |  |  | 0.942(0.697,1.273) | 0.696 | 0.925(0.680,1.256) | 0.616 |  | 0.841(0.625,1.133) | 0.256 | 0.836(0.614,1.138) | 0.255 |
| *Abbreviations: OR, odds ratio; NGT, normal glucose tolerance; T2DM, type 2 diabetes.*  Notes: *adjusted for age, gender, regular exercise, income, excessive drinking, smoking, hypertension, overweight. | | | | | | | | | | | | | |
