# Supplementary material for: UCP2 and UCP3 variants and gene-environment interaction associated with prediabetes and T2DM in a rural population: a case control study in China
Source: BMC Med Genet. 2018 Mar 12;19:43. doi: 10.1186/s12881-018-0554-4 (PMC5848510; doi:10.1186/s12881-018-0554-4)
Supplement: Supplementary file 4 — Table S4. Best gene-environment interaction models identified by GMDR. (DOCX 16 kb) [file 12881_2018_554_MOESM4_ESM.docx]

| **Table S4** Best gene-environment interaction models identified by GMDR | | | |
| --- | --- | --- | --- |
| Model | cross-validation consistency | testing accuracy | P |
| Prediabetes |  |  |  |
| overweight | 10/10 | 0.561 | **0.011** |
| overweight,rs1800849 | 6/10 | 0.527 | **0.011** |
| regular exercise, overweight, rs1800849 | 4/10 | 0.518 | 0.055 |
| T2DM |  |  |  |
| overweight | 10/10 | 0.609 | **0.001** |
| hypertension, overweight | 10/10 | 0.620 | **0.001** |
| hypertension, overweight,rs660339 | 4/10 | 0.611 | **0.011** |
| Notes: Bold denotes significant at *p*-value < 0.05. | | | |
